# Increased Levels of Genomic Instability and Mutations in Homologous Recombination Genes in Locally Advanced Rectal Carcinomas

**DOI:** 10.3389/fonc.2019.00395

**Published:** 2019-05-14

**Authors:** Luisa Matos do Canto, Simon J. Larsen, Bruna E. Catin Kupper, Maria Dirlei Ferreira de Souza Begnami, Cristóvam Scapulatempo-Neto, Annabeth Høgh Petersen, Mads M. Aagaard, Jan Baumbach, Samuel Aguiar, Silvia R. Rogatto

**Affiliations:** ^1^International Research Center — CIPE, A. C. Camargo Cancer Center, São Paulo, Brazil; ^2^Department of Clinical Genetics, Vejle Hospital, Vejle, Denmark; ^3^Department of Mathematics and Computer Science, University of Southern Denmark, Odense, Denmark; ^4^Department of Pelvic Surgery, A. C. Camargo Cancer Center, São Paulo, Brazil; ^5^Department of Pathology, A. C. Camargo Cancer Center, São Paulo, Brazil; ^6^Molecular Oncology Research Center, Barretos, Brazil; ^7^Diagnósticos da América, São Paulo, Brazil; ^8^TUM School of Life Sciences Weihenstephan, Technical University of Munich (TUM), Freising, Germany; ^9^Institute of Regional Health Research, University of Southern Denmark, Odense, Denmark; ^10^Danish Colorectal Cancer Center South, Vejle, Denmark

**Keywords:** rectal cancer, DNA copy number changes, neoadjuvant therapy, genomic instability, DNA repair deficiency

## Abstract

Pre-operative 5-fluoracil-based chemoradiotherapy (nCRT) is the standard treatment for patients with locally advanced rectal cancer (LARC). Patients with pathological complete response (pCR–0% of tumor cells in the surgical specimen after nCRT) have better overall survival and lower risk of recurrence in comparison with incomplete responders (pIR). Predictive biomarkers to be used for new therapeutic strategies and capable of stratifying patients to avoid overtreatment are needed. We evaluated the genomic profiles of 33 pre-treatment LARC biopsies using SNP array and targeted-next generation sequencing (tNGS). Based on the large number of identified genomic alterations, we calculated the genomic instability index (GII) and three homologous recombination deficiency (HRD) scores, which have been reported as impaired DNA repair markers. We observed high GII in our LARC cases, which was confirmed in 165 rectal cancer cases from TCGA. Patients with pCR presented higher GII compared with pIR. Moreover, a negative correlation between GII and the fraction of tumor cells remaining after surgery was observed (ρ = –0.382, *P* = 0.02). High HRD scores were detected in 61% of LARC, of which 70% were incomplete responders. Using tNGS (105 cancer-related genes, 13 involved in HR and 5 in mismatch repair pathways), we identified 23% of cases with mutations in HR genes, mostly in pIR cases (86% of mutated cases). In agreement, the analysis of the TCGA dataset (*N* = 145) revealed 21% of tumors with mutations in HR genes. The HRD scores were shown to be predictive of better response to PARP-inhibitors and platinum-based chemotherapy in breast and ovarian cancer. Our results suggest that the same strategy could be applied in a set of LARC patients with HRD. In conclusion, we identified high genomic instability in LARC, which was related to alterations in the HR pathway, especially in pIR. These findings suggest that patients with impaired HRD would clinically benefit from PARP-inhibitors and platinum-based therapy.

## Introduction

Several studies have demonstrated that colorectal cancer (CRC) is a heterogeneous disease and that rectal and colon tumors differ in many aspects (1). Among them, risk factors (gender, age, body mass index, dietary association), mutational profiling (most rectal cancers are negative for mutations in mismatch repair genes, while other genes are poorly explored), microsatellite instability (MSI is rare in rectal cancer) and CpG island methylator phenotype (CIMP is 0-low in rectal cancer) have been described (2, 3).

Chromosomal instability (CIN) and MSI are well-recognized mechanisms in CRC. CIN results from high levels of chromosomal mis-segregation, defects on telomere stability and/or DNA damage response (4). Although several approaches to quantify CIN have been described, the thresholds to consider CIN-positive are not standardized (5, 6).

Large DNA copy number alterations (named genomic scars) have been associated with the inability to repair DNA double strand breaks (DSB), which is the most hazardous type of DNA damage. The DSB are mostly repaired by non-homologous end joining (NHEJ) and homologous recombination (HR) repair mechanisms. The ability to predict HR deficiency (HRD), and consequently impaired DNA damage repair, can be determinant of response to chemotherapeutic agents, as reported in breast cancer (7–9). Scores used to capture large-scale transition (LST), telomeric allelic imbalance (tAI), and homologous recombination deficiency-loss of heterozygosity (HRD-LOH) have been used as tools to predict HRD (7–11). Overall, tumors with high scores are very likely to carry a deficient DNA damage repair system, resulting in a better response to platinum-based therapy (10).

The management of patients with locally advanced rectal carcinomas (LARC) with neoadjuvant 5-fluorouracil-based chemo and radiotherapy (nCRT) followed by total mesorectal excision has resulted in reduced locoregional recurrence and death by the disease. Cases achieving pathological complete response (pCR) to nCRT have shown lower rates of local and distant recurrences, and better survival compared to patients with incomplete response (pIR) (12). However, 70–90% of the patients present partial response and ~ 20% show resistance to treatment (13). In addition, the nCRT treatment results in significant morbidity, including long-term side effects and a high rate of clinical complications after surgery (14–18). Recent results from the German CAO/ARO/AIO-04 study showed that adding oxaliplatin to the current nCRT can result in improved disease-free survival (19). However, no molecular characterization was used to select the patients included in this strategy of treatment.

Although few studies described the genomic profile of LARC, the association of nCRT response with chromosomal imbalances and genomic instability has been poorly explored (20, 21). The first study reporting such association used comparative genomic hybridization (CGH) (22). The authors showed higher average of chromosomal imbalances in responders compared with non-responders. Subsequently, array-CGH data revealed higher, but not significant, chromosomal instability in resistant tumors (20, 23). Scores of HRD have not yet been reported in LARC, but an elevated chromosomal missegregation and loss of MRE11 function, a key player of the homologous recombination repair pathway, were associated with better pathological response to chemoradiotherapy in these patients (21).

Based on the current protocol used to treat LARC patients, it is mandatory to identify tumor inherent characteristics capable of stratifying patients according to response to therapy. Moreover, new therapeutic strategies associated with lower morbidity, recurrence rates and better overall survival are needed. Herein, we performed target enrichment for next generation sequencing and genome-wide SNP array in 33 LARC samples carefully selected aiming to verify the predictive value of chromosomal instability based in scores as biomarkers of nCRT response.

## Materials and Methods

### Patients

Clinical data from all patients with locally advanced rectal cancer admitted at the A. C. Camargo Cancer Center and Barretos Cancer Hospital, São Paulo Brazil, from 2006 to 2015, were carefully revised. Patients diagnosed with LARC by clinical (according to the AJCC 7th ed.) (24) and image evaluations referred to neoadjuvant treatment with continuous infusion of 5-fluouracil or oral capecitabine and radiotherapy (total dose of 50.4 Gy) followed by surgery were included in this study. In addition, the presence of previous cancer and/ or metastases at diagnosis was an exclusion criterion. From this cohort, a large number of patients were excluded due to incomplete clinical data, different treatment strategies or unavailable biopsies. According to these criteria, 33 unrelated patients were included in this study. The biopsy specimens were obtained during colonoscopy prior to pre-operative chemoradiotherapy and evaluated by two specialized pathologists (MDFSB and CSN). Comprehensive clinical, pathological and epidemiological data were obtained from the medical records. The histopathological analysis was revised and a tumor area with more than 70% tumor fraction was selected for molecular evaluation. The Human Research Ethics Committee from both Institutions approved the study (Protocols 1884/14 and 1030/2015, respectively). All patients provided written informed consent prior to sample collection.

### Classification of Response

Pathological complete response (pCR; ypT0N0) or pathological incomplete response (pIR) groups were selected according to the absence or presence of reminiscent viable tumor cells in the surgical specimens, respectively. Tumor regression grade (TRG) was scored according to Dworak and Mandard system with modifications (25). The cases were assigned into four categories according to the percentage of viable tumor cells: Grade 0: complete tumor regression (0% of residual tumor cells); Grade I: subtotal tumor regression (< 25% of viable tumor cells); Grade II: partial tumor regression (25–50% of viable tumor cells); Grade III: minimal tumor regression (> 50% of viable tumor cells). Samples with complete tumor regression and showing lymph nodes (ypT0N+) were classified as Grade II (25).

### Genomic Profiling

DNA was extracted from frozen tumor specimens using phenol-chloroform-isoamyl alcohol (25:24:1) solution. Genomic imbalances were assessed using the CytoScan HD array (Affymetrix, Santa Clara, CA, USA), according to the manufacturer recommendations. The scanning (Scanner 3000 7G, Affymetrix) and [.CEL] files were generated (Affymetrix® GeneChip® Command Console® Software v.4.0) followed by data analysis Chromosome Analysis Suite (ChAS v.3.1, Affymetrix). Significant alterations were considered with at least 25 probes altered for losses, 50 for gains, and a minimum of 5 Mb for cnLOH (copy-neutral loss of heterozygosity), as previously described (26). Regions with common variants (>1% of the population study) identified using the Database of Genomic Variants (DGV, http://dgv.tcag.ca/dgv/app/home, updated in May 2016) and Affymetrix Database of Variants (composed by 2,421 health individuals evaluated with the Cytoscan HD array) were excluded from subsequent analyses.

### Genomic Instability Index and Homologous Recombination Deficiency Scores

The fraction of the genome affected by copy-number changes (Genomic Instability Index, GII) was calculated for each individual case, as previously described (27). The median GII values were compared according to therapy response (pCR vs. pIR, and TRG). Considering that the fraction of tumor cells remaining in the surgical specimen after nCRT is a surrogate of treatment response, we evaluated the correlation between the GII and the % of viable tumor cells identified after surgery.

The homologous recombination deficiency scores (LST, tAI, and HRD-LOH) were calculated as previously described (28). In summary, LST indicates the number of chromosomal breaks between adjacent regions of at least 10 Mb (high: >15 in diploid tumors and > 20 in polyploidy tumors); tAI is referred as the number of subtelomeric regions with allelic imbalance that start beyond the centromere and extended to the telomere region (high: > median value). HRD-LOH indicates the number of LOH regions larger than 15 Mb and shorter than the whole chromosome (high: >10).

### Target Enrichment—Next Generation Sequencing (tNGS)

Targeted NGS was performed using a 105 cancer-related genes panel (all exons, 3′UTR and 5′UTR) including 13 genes involved in the HR pathway and five MMR genes (SureSelectXT Custom Panel, Agilent Technologies, Inc., Santa Clara, CA) (Table S1). The libraries were prepared using SureSelectQXT Library Prep Kit (Agilent) according to manufacturer's instructions and sequenced on NextSeq 550 (Illumina, San Diego, CA). For each patient, 2 μL of genomic DNA (25 ng/μL) was used for enzymatic fragmentation. Library amplification was performed using Herculase II Fusion DNA Polymerase (Agilent) and the PCR product was purified using the Agencourt AMPureXP purification bead system (Beckman Coulter; Pasadena, CA). The targeted DNA was captured using streptavidin-coated magnetic beads (Dynabeads MyOne Streptavidin T1; Thermo Fisher Scientific, Waltham, MA) followed by indexing (SureSelectQXT P7 and P5 dual indexing primers). The analysis of amplified indexed library DNA was performed using High Sensitivity D1000 ScreenTape (on Agilent TapeStation). Two samples were excluded (DNA quantity and quality) and 31 were multiplexed into 1.4 pM pool and loaded onto the NextSeq 550 (Illumina).

### tNGS Data Analysis

The raw sequencing reads demultiplexing and FASTQ files were obtained, with the resulting reads being mapped to the UCSC human genome reference build 19 using the BWA alignment algorithm (29). Variant calling and quality filtering were performed with GATK (30). Variant annotation was primarily done using ANNOVAR (31), GATK and SnpEff (32). Ingenuity software (Qiagen, Hilden, Germany) was used to classify the variants according to the American College of Medical Genetics and Genomics (ACMG) (33). The pipeline for variant filtering is represented in Figure S1. Shortly, benign or likely benign variants (ACMG) and those with frequency > 0.01 in the GAD (34) and/or in the ExAC (35) were excluded. The remaining variants were manually curated using the Genome Browse software (Golden Helix Inc., Bozeman, MT). The variants mapped in homopolymer regions were excluded and a new filtering was applied to exclude variants of uncertain significance (VUS, classified by ACMG) with CADD score < 3.0.

### Cross-Validation Analysis

Genome wide SNP6 segmented somatic copy number alterations data from 165 rectal cancers were retrieved from TCGA (available at Xena Browser, accessed on March 2018: https://tcga.xenahubs.net/download/TCGA.READ.sampleMap/SNP6_nocnv_genomicSegment.gz). Segments were filtered (log2 ratio of 0.2) and GII was calculated as described above (27). The mutational profile of 145 of 165 rectal cancer cases were available (cBioPortal; accessed on April 2018: http://www.cbioportal.org/) and used to evaluate the mutational status of 23 MMR genes (KEGG: hsa03430) and 41 HR genes (KEGG: hsa03440) in association to GII.

### Statistical Analysis

Statistical analyses were performed using the SPSS (SPSS 24 Inc, Chicago, IL, USA). Mann-Whitney U and Fisher exact test were applied to investigate association among the clinical parameters, GII, HRD-scores and nCRT response. The non-parametric Kruskall-Wallis test was applied to compare multiple groups. The Spearman correlation coefficient (ρ) was used to assess the relationship between two variables. A comparison of segmented CNAs data between groups according to the response to nCRT was performed using the CoNVaQ web tool (36). Computation of empirical *p*-values for permutation testing was performed for finding a matching region of the same length or longer, when the size of each group is preserved, but the samples are randomly distributed among groups (36).

## Results

### Patient Characteristics and Response to nCRT

Clinical parameters and response to nCRT of patients included in this study are summarized in Table 1. Eleven (33%) patients achieved pCR (or TRG Grade 0) and 22 (67%), pIR (TRG 1: 10, TRG 2: 9, and TRG 3: 3). The analysis of clinical features and pathological response showed no statistical significance (data not shown).

**Table 1:** Clinical and histopathological characteristics of 33 locally advanced rectal cancer patients included in this study.

**Characteristics**	***N* = 33**
Median age at diagnosis (range, years)	56 (26–80)
**Gender**
Male	20
Female	13
**Response to neoadjuvant therapy**
pCR	11
pIR	22
**Tumor regression grade**
TRG0	11
TRG1	10
TRG2	9
TRG3	3
**cT stage**
T2	6
T3	25
T4	2
**cN stage**
N0	10
N+	23
**ypT stage**
T0	12
T1	1
T2	11
T3	7
T4	2
**ypN stage**
N0	27
N+	6
**Metastasis**	5
**Cell differentiation**
Well	8
Moderate	24
NA	1

*TNM, tumor-node-metastasis (AJCC 7th edition); pCR, pathological complete response; pIR, pathological incomplete response; TRG, Tumor Regression Grade; NA, not available*.

### Copy Number Alterations and Chromosomal Instability Analysis

A total of 1,442 CNAs was detected including 880 gains, 442 losses and 120 cnLOH (596 in 11 pCR; 846 in 22 pIR), ranging from 1 to 161 per case. Gains of 7p, 8q, 13q, and 20q and losses of 8p, 14, 17p, and 18 were frequently detected. The statistical model of CoNVaQ (36) pointed out 45 significant chromosomal regions mapped at chromosomes 4, 8, 19, 20, and 21 (*p* < 0.05) associated with therapeutic response (Table S2). Loss of chromosome 21 (start 35020505—end 40248388) was found only in pIR cases (36.4%) (permutation *p*-value > 0.29).

**Figure 1:**
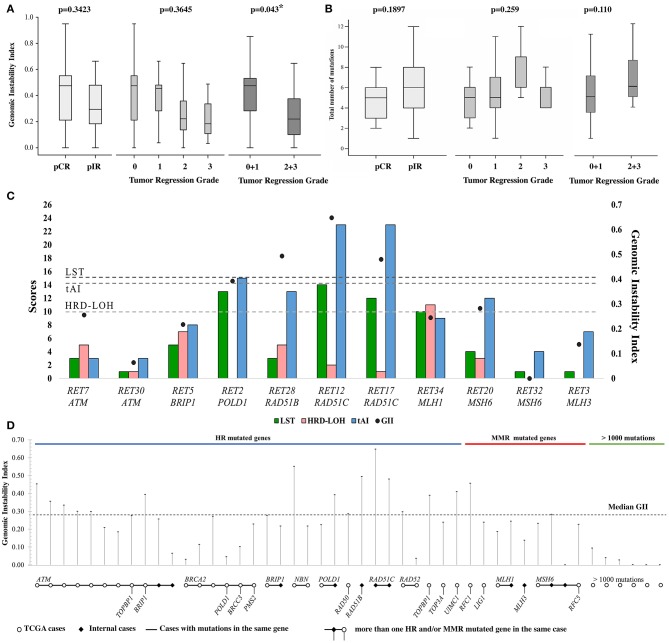
**(A)** Distribution of the Genomic Instability Index (GII) and **(B)** the number of mutations found in rectal cancer samples according to pathological response (pCR: complete response or pIR: incomplete) to neoadjuvant chemoradiotherapy. Cases were also categorized into tumor regression grade (TRG) and grouped in TRG 0 + 1 and TRG 2 + 3. **(C)** Homologous recombination deficiency scores (LST, LOH, and tAI) and GII of samples with mutation in DNA repair genes. **(D)** GII of each TCGA and internal cases carrying mutation in DNA damage repair pathways (homologous recombination and mismatch repair) genes. Samples carrying mutation in the same gene are shown connected by a line, additional genes are shown below each sample. Samples with >1000 mutations are indicated, and specific genes were not taken into consideration for these cases. Five of 14 cases from TCGA with mutation in ATM presented >1000 mutations, and nine of them are represented.

**Figure 2:**
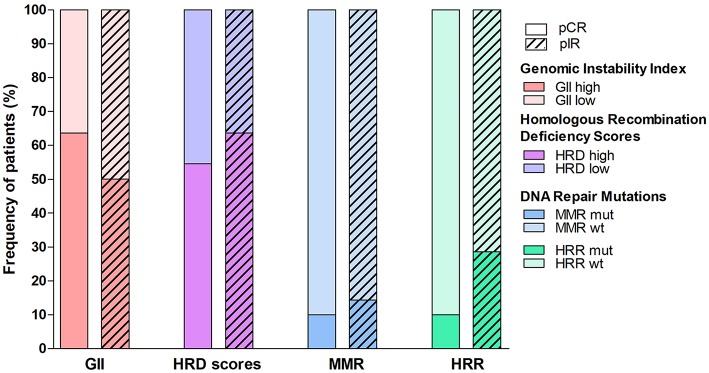
Proportion of locally advanced rectal cases with pathological complete response (pCR) or incomplete response (pIR) to neoadjuvant therapy according to the genomic features evaluated in this study. Patients were divided into high or low levels of Genomic Instability Index (GII), elevated values of at least one of the three scores of Homologous Recombination Repair Deficiency (HRD scores), and mutation in genes related to DNA damage repair (mismatch repair-MMR or HR repair-HRR).

A similar GII distribution was found comparing our LARC cases (*N* = 33, median = 0.358) with the TCGA dataset (*N* = 165, median = 0.284) (*P* > 0.05). The GII median value was used as threshold (*N* = 198, median = 0.286). Eighteen (55%; 11/22 pIR and 7/11 pCR, Figure 2) and 81 (49%) rectal tumors from our dataset and TCGA, respectively, presented high GII levels. Although statistically non-significant, pCR presented higher median GII (0.475) compared to incomplete responders (0.294). Similar analysis using the TRG revealed an opposite trend between the median GII and TRG. A significant difference was found grouping TRG 0+1 and TRG 2+3 (*P* = 0.043) (Figure 1A). A negative correlation between GII and the fraction of tumor cells remaining after surgery was observed (ρ = −0.382, *P* = 0.02). Information for TCGA cases regarding the response to nCRT was not available, preventing further comparisons.

### Homologous Recombination Deficiency Scores

The HRD score on median values of tAI, LST and HRD-LOH were 14, 6, and 2, respectively. Using median values > 14.0 as cut-off for tAI, 14 of 22 pIR and 4 of 11 pCR presented high scores. Two pCR cases showed high LST (>15 or >20 for diploid or polyploid tumors) and five cases (3pIR and 2pCR) presented HRD-LOH scores higher than 10. Potential deficiency in HR pathway (at least one high score) was found in 55% of pCR and 64% of pIR cases (Figure 2). No significant differences were found according to the nCRT and clinical features (Table 2, Figure S2).

**Table 2:** The number of cases with high Genomic Instability Index (GII) and predicted homologous recombination deficiency (HRD) based on three scores (tAI, LOH, and LST).

**Score**	**Criteria**	**pCR**	**pIR**	***P*-value**
GII	High > 0.286	7	11	0.712
	Low < 0.286	4	11	
nTAI	>14	4	14	0.163
	< 14	7	8	
HRD-LOH	>10	3	2	0.304
	< 10	8	20	
LST	>15 if 2N or >20 if 4N	2	0	0.118
	< 15 if 2N or < 20 if 4N	9	22	

*GII, Genomic Instability Index; nTAI, number of telomere allelic imbalance; HRD-LOH, Loss of heterozygosity score; LST, Large Scale Transition score; calculated as described in Material and Methods session*.

The Spearman correlation coefficient between each pair of scores was significant for LST and tAI (ρ = 0.720, *p* < 0.001) scores. Significant correlation between GII and tAI was detected for pIR cases (ρ = 0.470, *p* = 0.02), while no correlation was observed between the scores and GII for pCR cases. HRD scores and GII for each case are depicted in Table S3.

### Mutational Profile of Rectal Cancer

Targeted-NGS analysis of 31 cases revealed 161 unique variants in 51 genes, with an average of five mutations per case in pCR, and six in pIR (Figure 1B, Table S4). Thirteen genes were mutated regardless of response to therapy, including *TP53* (84%), *APC* (81%) and *KRAS* (45%). Eight genes were mutated exclusively in pCR cases and 30 genes in pIR. The frequency of mutated genes was similar to the TCGA data (Fisher's, *P* > 0.05), except for five genes with higher frequency in our cases (*FLT1, FLT4, MMP1, RAD51C, WNT1*) (Figure S3).

Seven cases (23%) presented HR genes mutation, including *ATM* (2pIR), *BRIP1* (1pIR), *POLD1* (1pIR), *RAD51B* (1pCR), and *RAD51C* (2pIR). Four cases (13%) harbored MMR gene mutations: *MLH1* (1pCR), *MLH3* (1pIR) and *MSH6* (2pIR) (Figure 2). Similarly, the TCGA dataset (*N* = 145) presented 21% of tumors with HR gene mutations and 10% with MMR gene mutations. Four cases with HR gene mutations also showed high HRD scores (Figure 1C).

Next generation sequencing data from 145 rectal cancer retrieved from TCGA were used to evaluate the mutational status of 23 MMR genes (KEGG: hsa03430) and 41 HR genes (KEGG: hsa03440) in association to GII. Cases with GII higher than the threshold (median of 0.286) presented mutations almost exclusively in HR genes. Only one case carried MMR mutation (RFC1). Low GII values (median = 0.01) were detected in cases presenting more than 1,000 mutations. Six of 14 ATM mutated cases had high GII (median = 0.35), while five, that were also hypermutated cases (>1,000 including MMR genes), presented low GII (median = 0.01). The GII values from TCGA data and our internal dataset according to the mutation status of HR/MMR genes are depicted in Figure 1D.

### Correlation of Genomic Alterations and Response to nCRT

The genomic variables (GII, HRD scores and mutational profile) were categorized in high and low or mutated and wild type (WT) to compare pCR with pIR cases (Figure 2). This analysis revealed slight difference between the two groups. The pCR cases presented increased proportion of high GII, but lower rate of cases with at least one high HRD score and mutations in MMR and HR genes compared with pIR group.

## Discussion

Genomic alterations and response to therapy in LARC are insufficiently understood and explored. In our study, LARC cases presented a high level of chromosomal instability recapitulating the most common alterations previously reported in literature (20, 23, 37). In addition, loss of chromosome 21, where *RUNX1* is mapped, was detected exclusively in pIR cases (36.4%). This tumor suppressor encodes a transcription factor downregulated in several cancers (38).

We calculated the medium values of the genomic instability index (GII) based on the findings obtained in the cohort of 165 rectal cancer from TCGA and our set of 33 LARC (median GII of 0.286). Comparing with the pan-cancer analysis of genomic scar signatures, this is higher than described in prostate adenocarcinoma (0.081) and lower than breast (0.435) and ovarian serous carcinoma (0.574) (TCGA data) (28).

To investigate the presence of impaired DNA damage repair, three scores used to predict HRD were evaluated by capturing tAI, HRD-LOH and LST (7, 10, 11). Interestingly, high median scores were detected for tAI (14), but low for LST (6) and HRD-LOH (2). Marquard et al. (28) evaluated HRD scores in 15 different tumor types, including colorectal adenocarcinomas. Based on the average of these three scores, colon cancer was ranked at 10th position while our LARC was at 7th position (below breast cancer). High HRD scores were associated to increased sensitivity to platinum-based chemotherapy and PARP-inhibitors in breast and ovarian cancer (9). A considerable subset of our cases presented high HRD scores (61%; of which 70% were pIR) suggesting that these patients could benefit from platinum-based therapy.

The correlation among the three HRD scores revealed a positive significant value between tAI and LST (ρ = 0.711). In agreement, Marquard et al. showed a correlation between these two scores (ρ > 0.7 in 8 of 15 cancer types) and that HRD-LOH presented lower correlation with tAI and LST in 13 of 15 tumors (28). Overall, no significant correlation between GII and these three scores was found. However, GII was positively correlated with tAI (ρ = 0.470, *p* = 0.02) in pIR cases, suggesting that GII in rectal cancer quantify different aspects of CNA, being probably associated with HRD. Alternative mechanisms could be involved in pCR cases. In fact, the correlation between GII and HRD scores in ovarian cancer was much lower than the median value for all cancer types and more similar to our findings (28).

Interestingly, mutations in genes involved in HR repair were detected in 23% of our LARC cases and in 21% of TCGA cohort, respectively. Six of the seven cases with mutations in one of these genes (*ATM, BRIP1, POLD1, RAD51B, RAD51C*) presented incomplete response to therapy. We detected an elevated tAI score and GII in two cases presenting *RAD51C* mutation. In cancer cells, mutated *RAD51C* recruits non-homologous end joining (NHEJ) proteins, which correlates to higher genomic instability due to error-prone repair. Deficiency of RAD51C confers enhanced sensitivity to Poly (ADP-ribose) Polymerase 1 (PARP1) inhibitors in combination with radiation, which can be used as an alternative synergistic treatment approach for 5-FU resistant tumors (39). The synthetic lethality achieved with PARP1 inhibitors was also observed in pancreatic ductal adenocarcinoma cells with *ATM* mutation. Two LARC cases with pIR presented *ATM* mutations. ATM deficiency promotes genomic instability by blunting DNA damage repair and leading to complex genomic aberration such as chromothripsis (40), which we detected in one *ATM* mutated case (chromosome 20).

The mutational profiling of 145 cases from TCGA revealed *ATM* mutation in 10% of the cases; 43% of them presented high GII. Nonetheless, this correlation was stronger when excluding cases with more than 1,000 mutations (78% with high GII). All five *ATM* mutated cases that also carried more than 1,000 mutations, including in MMR genes, had very low GII. Mutations found in MMR and HR genes in such cases can be a consequence of their hypermutated phenotype and should not be considered for an association with GII.

Although the initial results of clinical trials evaluating the addition of oxaliplatin to the conventional 5-FU based nCRT regimen have shown no additional benefits (41), an increased disease-free survival was observed after long-term follow-up (19). Compared with older patients (aged ≥ 70 years), those under 60 years of age presented a reduced number of recurrences, metastasis and better overall survival (42). However, the HR pathway was not assessed, and this analysis could be predictive of response to oxaliplatin.

No association between the number of mutations or a specific mutated gene and the complete response to nCRT was observed. The most frequently mutated genes were *TP53, APC*, and *KRAS*. Wild type *TP53* was associated with a good response to nCRT (Risk Ratio = 1.30, 95% CI = 1.14–1.49, *P* < 0.001) in a meta-analysis with 1,830 rectal cancer patients from 30 studies (43). However, the lack of standard treatment regimen, the definition of methods, their quantification, and outcome may lead to bias in the meta-analysis. The predictive power of *KRAS* in the nCRT response was also reported, in which codon 13 mutation showed association to a worse response to therapy (44, 45). Furthermore, the small number of LARC evaluated in several studies, including our own, make the use of these mutations as predictive biomarkers still controversial.

Among the genes mutated only in our pCR cases, two members of the human ATP-binding cassete (ABC) transporter superfamily present compelling evidence of a role in chemoresistance. *ABCG2* is expressed in gastrointestinal tissue and its inhibition was shown to increase the sensitivity to 5-FU *in vitro* and *in vivo* (46). Inhibition of *ABCC1* expression has been reported as leading to 5-FU enhanced response in CRC cell lines (47). This gene is transcriptionally regulated by MYC, which was also found mutated in our pCR case (48). In breast cancer and glioblastoma cell lines, the downregulation of MYC protein resulted in suppression of ABCC1, being a putative mechanism to sensitize tumors to chemotherapy (48). Although neoadjuvant treatment information from the TCGA cohort is unavailable, a comparison with our data revealed similar frequencies of these ABC gene mutations (*ABCC1*: 3% in our data and 6%: TCGA; *ABCG2*: 3% in both).

Although we presented new and relevant data, a set of limitations could be pointed out including the small sample size, a challenge also faced by the previous large-scale genomic studies in LARC (20, 22, 23). Furthermore, the use of single pre-treatment biopsies precludes the evaluation of intra-tumoral heterogeneity, already described in a set of rectal tumors (49). As tumors can be an assembly of different subclones, intra-tumor heterogeneity can also play a role in tumor response to therapy. However, the implication of heterogeneity in the prediction and prognosis of rectal cancer has yet to be explored.

In conclusion, we evaluated the genomic profile of LARC in association with the response to nCRT and quantified the observed high chromosomal instability into HRD scores and GII. Similar to breast and ovarian cancer, the HRD scores were high in LARC and further studies are necessary to evaluate its association with the sensitivity to platinum-based chemotherapy and PARP-inhibitors. The assessment of HRD can be used to select patients for neoadjuvant chemotherapy strategy. A set of tumors with high genomic instability (GII) can be explained by mutations in genes involved in DNA repair by homologous recombination. Alteration in these genes was observed in 23% of our cases and in 21% of the TCGA dataset, revealing promising results as new targets for therapy alone or in combination with the current nCRT.

## Ethics Statement

This study was carried out in accordance with the recommendations of Human Research Ethics Committees from A. C. Camargo Cancer Center and Barretos Cancer Hospital, São Paulo Brazil. All subjects gave written informed consent in accordance with the Declaration of Helsinki. The protocol was approved by both Committees (Protocols 1884/14 and 1030/2015, respectively).

## Author Contributions

SR and SA conceived and designed the study. MB and CS-N performed histopathological evaluation. SA, BC, LC, and SR selected the cases and obtained the clinical data. LC conducted the experiments. LC, SL, JB, AP, and MA analyzed the data. LC and SR wrote and edited the manuscript. All authors read and approved the final version of the manuscript.

### Conflict of Interest Statement

The authors declare that the research was conducted in the absence of any commercial or financial relationships that could be construed as a potential conflict of interest.
