# Supplementary material for: Increased Levels of Genomic Instability and Mutations in Homologous Recombination Genes in Locally Advanced Rectal Carcinomas
Source: Front Oncol. 2019 May 14;9:395. doi: 10.3389/fonc.2019.00395 (PMC6527873; doi:10.3389/fonc.2019.00395)
Supplement: Supplementary file 1 [file Table_1.DOCX]

Supplementary Material

# Supplementary Tables

**Supplementary Table S1**. Cancer related genes, used for target enrichment and next-generation sequencing of rectal tumors, annotated using UniProt [1].

| **Genes** | **Protein names** |
| --- | --- |
| *ABCC1* | Multidrug resistance-associated protein 1 (ATP-binding cassette sub-family C member 1) (Leukotriene C (4) transporter) (LTC4 transporter) |
| *ABCG2* | ATP-binding cassette sub-family G member 2 (Breast cancer resistance protein) (CDw338) (Mitoxantrone resistance-associated protein) (Placenta-specific ATP-binding cassette transporter) (Urate exporter) (CD antigen CD338) |
| *AKT1* | RAC-alpha serine/threonine-protein kinase (EC 2.7.11.1) (Protein kinase B) (PKB) (Protein kinase B alpha) (PKB alpha) (Proto-oncogene c-Akt) (RAC-PK-alpha) |
| *ALK* | ALK tyrosine kinase receptor (EC 2.7.10.1) (Anaplastic lymphoma kinase) (CD antigen CD246) |
| *APC* | Adenomatous polyposis coli protein (Protein APC) (Deleted in polyposis 2.5) |
| *AR* | Androgen receptor (Dihydrotestosterone receptor) (Nuclear receptor subfamily 3 group C member 4) |
| *AREG* | Amphiregulin (AR) (Colorectum cell-derived growth factor) (CRDGF) |
| *ARF1* | ADP-ribosylation factor 1 |
| *ATM** | Serine-protein kinase ATM (EC 2.7.11.1) (Ataxia telangiectasia mutated) (A-T mutated) |
| *BARD1** | BRCA1-associated RING domain protein 1 (BARD-1) (EC 2.3.2.27) (RING-type E3 ubiquitin transferase BARD1) |
| *BMPR1A* | Bone morphogenetic protein receptor type-1A (BMP type-1A receptor) (BMPR-1A) (EC 2.7.11.30) (Activin receptor-like kinase 3) (ALK-3) (Serine/threonine-protein kinase receptor R5) (SKR5) (CD antigen CD292) |
| *BRAF* | Serine/threonine-protein kinase B-raf (EC 2.7.11.1) (Proto-oncogene B-Raf) (p94) (v-Raf murine sarcoma viral oncogene homolog B1) |
| *BRCA1** | Breast cancer type 1 susceptibility protein (EC 2.3.2.27) (RING finger protein 53) (RING-type E3 ubiquitin transferase BRCA1) |
| *BRCA2** | Breast cancer type 2 susceptibility protein (Fanconi anemia group D1 protein) |
| *BRIP1** | Fanconi anemia group J protein (Protein FACJ) (EC 3.6.4.13) (ATP-dependent RNA helicase BRIP1) (BRCA1-associated C-terminal helicase 1) (BRCA1-interacting protein C-terminal helicase 1) (BRCA1-interacting protein 1) |
| *CCND1* | G1/S-specific cyclin-D1 (B-cell lymphoma 1 protein) (BCL-1) (BCL-1 oncogene) (PRAD1 oncogene) |
| *CDH1* | Cadherin-1 (CAM 120/80) (Epithelial cadherin) (E-cadherin) (Uvomorulin) (CD antigen CD324) [Cleaved into: E-Cad/CTF1; E-Cad/CTF2; E-Cad/CTF3] |
| *CDKN2A* | Cyclin-dependent kinase inhibitor 2A (Cyclin-dependent kinase 4 inhibitor A) (CDK4I) (Multiple tumor suppressor 1) (MTS-1) (p16-INK4a) (p16-INK4) (p16INK4A) |
| *CHEK2* | Serine/threonine-protein kinase Chk2 (EC 2.7.11.1) (CHK2 checkpoint homolog) (Cds1 homolog) (Hucds1) (hCds1) (Checkpoint kinase 2) |
| *COL11A1* | Collagen alpha-1(XI) chain |
| *CTNNB1* | Catenin beta-1 (Beta-catenin) |
| *DICER1* | Endoribonuclease Dicer (EC 3.1.26.3) (Helicase with RNase motif) (Helicase MOI) |
| *DNMT3A* | DNA (cytosine-5)-methyltransferase 3A (Dnmt3a) (EC 2.1.1.37) (DNA methyltransferase HsaIIIA) (DNA MTase HsaIIIA) (M.HsaIIIA) |
| *DNMT3B* | DNA (cytosine-5)-methyltransferase 3B (Dnmt3b) (EC 2.1.1.37) (DNA methyltransferase HsaIIIB) (DNA MTase HsaIIIB) (M.HsaIIIB) |
| *EGF* | Pro-epidermal growth factor (EGF) [Cleaved into: Epidermal growth factor (Urogastrone)] |
| *EGFR* | Epidermal growth factor receptor (EC 2.7.10.1) (Proto-oncogene c-ErbB-1) (Receptor tyrosine-protein kinase erbB-1) |
| *EPCAM* | Epithelial cell adhesion molecule (Ep-CAM) (Adenocarcinoma-associated antigen) (Cell surface glycoprotein Trop-1) (Epithelial cell surface antigen) (Epithelial glycoprotein) (EGP) (Epithelial glycoprotein 314) (EGP314) (hEGP314) (KS 1/4 antigen) (KSA) (Major gastrointestinal tumor-associated protein GA733-2) (Tumor-associated calcium signal transducer 1) (CD antigen CD326) |
| *ERBB2* | Receptor tyrosine-protein kinase erbB-2 (EC 2.7.10.1) (Metastatic lymph node gene 19 protein) (MLN 19) (Proto-oncogene Neu) (Proto-oncogene c-ErbB-2) (Tyrosine kinase-type cell surface receptor HER2) (p185erbB2) (CD antigen CD340) |
| *ERBB3* | Receptor tyrosine-protein kinase erbB-3 (EC 2.7.10.1) (Proto-oncogene-like protein c-ErbB-3) (Tyrosine kinase-type cell surface receptor HER3) |
| *ERBB4* | Receptor tyrosine-protein kinase erbB-4 (EC 2.7.10.1) (Proto-oncogene-like protein c-ErbB-4) (Tyrosine kinase-type cell surface receptor HER4) (p180erbB4) [Cleaved into: ERBB4 intracellular domain (4ICD) (E4ICD) (s80HER4)] |
| *EREG* | Proepiregulin [Cleaved into: Epiregulin (EPR)] |
| *ESR1* | Estrogen receptor (ER) (ER-alpha) (Estradiol receptor) (Nuclear receptor subfamily 3 group A member 1) |
| *EZH2* | Histone-lysine N-methyltransferase EZH2 (EC 2.1.1.43) (ENX-1) (Enhancer of zeste homolog 2) (Lysine N-methyltransferase 6) |
| *FBXW7* | F-box/WD repeat-containing protein 7 (Archipelago homolog) (hAgo) (F-box and WD-40 domain-containing protein 7) (F-box protein FBX30) (SEL-10) (hCdc4) |
| *FGF1* | Fibroblast growth factor 1 (FGF-1) (Acidic fibroblast growth factor) (aFGF) (Endothelial cell growth factor) (ECGF) (Heparin-binding growth factor 1) (HBGF-1) |
| *FGFR1* | Fibroblast growth factor receptor 1 (FGFR-1) (EC 2.7.10.1) (Basic fibroblast growth factor receptor 1) (BFGFR) (bFGF-R-1) (Fms-like tyrosine kinase 2) (FLT-2) (N-sam) (Proto-oncogene c-Fgr) (CD antigen CD331) |
| *FGFR2* | Fibroblast growth factor receptor 2 (FGFR-2) (EC 2.7.10.1) (K-sam) (KGFR) (Keratinocyte growth factor receptor) (CD antigen CD332) |
| *FLT1* | Vascular endothelial growth factor receptor 1 (VEGFR-1) (EC 2.7.10.1) (Fms-like tyrosine kinase 1) (FLT-1) (Tyrosine-protein kinase FRT) (Tyrosine-protein kinase receptor FLT) (FLT) (Vascular permeability factor receptor) |
| *FLT3* | Receptor-type tyrosine-protein kinase FLT3 (EC 2.7.10.1) (FL cytokine receptor) (Fetal liver kinase-2) (FLK-2) (Fms-like tyrosine kinase 3) (FLT-3) (Stem cell tyrosine kinase 1) (STK-1) (CD antigen CD135) |
| *FLT4* | Vascular endothelial growth factor receptor 3 (VEGFR-3) (EC 2.7.10.1) (Fms-like tyrosine kinase 4) (FLT-4) (Tyrosine-protein kinase receptor FLT4) |
| *FOXL2* | Forkhead box protein L2 |
| *FOXO1* | Forkhead box protein O1 (Forkhead box protein O1A) (Forkhead in rhabdomyosarcoma) |
| *GALNT12* | Polypeptide N-acetylgalactosaminyltransferase 12 (EC 2.4.1.41) (Polypeptide GalNAc transferase 12) (GalNAc-T12) (pp-GaNTase 12) (Protein-UDP acetylgalactosaminyltransferase 12) (UDP-GalNAc:polypeptide N-acetylgalactosaminyltransferase 12) |
| *GNA11* | Guanine nucleotide-binding protein subunit alpha-11 (G alpha-11) (G-protein subunit alpha-11) (Guanine nucleotide-binding protein G(y) subunit alpha) |
| *GNAQ* | Guanine nucleotide-binding protein G(q) subunit alpha (Guanine nucleotide-binding protein alpha-q) |
| *GPS1/CSN1* | COP9 signalosome complex subunit 1 (SGN1) (Signalosome subunit 1) (G protein pathway suppressor 1) (GPS-1) (JAB1-containing signalosome subunit 1) (Protein MFH) |
| *GREM1* | Gremlin-1 (Cell proliferation-inducing gene 2 protein) (Cysteine knot superfamily 1, BMP antagonist 1) (DAN domain family member 2) (Down-regulated in Mos-transformed cells protein) (Increased in high glucose protein 2) (IHG-2) |
| *HGF* | Hepatocyte growth factor (Hepatopoietin-A) (Scatter factor) (SF) [Cleaved into: Hepatocyte growth factor alpha chain; Hepatocyte growth factor beta chain] |
| *HRAS* | GTPase HRas (H-Ras-1) (Ha-Ras) (Transforming protein p21) (c-H-ras) (p21ras) [Cleaved into: GTPase HRas, N-terminally processed] |
| *HSPH1* | Heat shock protein 105 kDa (Antigen NY-CO-25) (Heat shock 110 kDa protein) |
| *IDH1* | Isocitrate dehydrogenase [NADP] cytoplasmic (IDH) (EC 1.1.1.42) (Cytosolic NADP-isocitrate dehydrogenase) (IDP) (NADP(+)-specific ICDH) (Oxalosuccinate decarboxylase) |
| *IDH2* | Isocitrate dehydrogenase [NADP], mitochondrial (IDH) (EC 1.1.1.42) (ICD-M) (IDP) (NADP(+)-specific ICDH) (Oxalosuccinate decarboxylase) |
| *IGF1* | Insulin-like growth factor I (IGF-I) (Mechano growth factor) (MGF) (Somatomedin-C) |
| *IGF1R* | Insulin-like growth factor 1 receptor (EC 2.7.10.1) (Insulin-like growth factor I receptor) (IGF-I receptor) (CD antigen CD221) [Cleaved into: Insulin-like growth factor 1 receptor alpha chain; Insulin-like growth factor 1 receptor beta chain] |
| *JAK1* | Tyrosine-protein kinase JAK1 (EC 2.7.10.2) (Janus kinase 1) (JAK-1) |
| *JUN* | Transcription factor AP-1 (Activator protein 1) (AP1) (Proto-oncogene c-Jun) (V-jun avian sarcoma virus 17 oncogene homolog) (p39) |
| *KDM4C* | Lysine-specific demethylase 4C (EC 1.14.11.-) (Gene amplified in squamous cell carcinoma 1 protein) (GASC-1 protein) (JmjC domain-containing histone demethylation protein 3C) (Jumonji domain-containing protein 2C) |
| *KIT* | Mast/stem cell growth factor receptor Kit (SCFR) (EC 2.7.10.1) (Piebald trait protein) (PBT) (Proto-oncogene c-Kit) (Tyrosine-protein kinase Kit) (p145 c-kit) (v-kit Hardy-Zuckerman 4 feline sarcoma viral oncogene homolog) (CD antigen CD117) |
| *KRAS* | GTPase KRas (K-Ras 2) (Ki-Ras) (c-K-ras) (c-Ki-ras) [Cleaved into: GTPase KRas, N-terminally processed] |
| *MAP2K1* | Dual specificity mitogen-activated protein kinase kinase 1 (MAP kinase kinase 1) (MAPKK 1) (MKK1) (EC 2.7.12.2) (ERK activator kinase 1) (MAPK/ERK kinase 1) (MEK 1) |
| *MEN1* | Menin |
| *MET* | Hepatocyte growth factor receptor (HGF receptor) (EC 2.7.10.1) (HGF/SF receptor) (Proto-oncogene c-Met) (Scatter factor receptor) (SF receptor) (Tyrosine-protein kinase Met) |
| *MLH1*** | DNA mismatch repair protein Mlh1 (MutL protein homolog 1) |
| *MLH3*** | DNA mismatch repair protein Mlh3 (MutL protein homolog 3) |
| *MMP1* | Interstitial collagenase (EC 3.4.24.7) (Fibroblast collagenase) (Matrix metalloproteinase-1) (MMP-1) [Cleaved into: 22 kDa interstitial collagenase; 27 kDa interstitial collagenase] |
| *MMP12* | Macrophage metalloelastase (MME) (EC 3.4.24.65) (Macrophage elastase) (ME) (hME) (Matrix metalloproteinase-12) (MMP-12) |
| *MMP13* | Collagenase 3 (EC 3.4.24.-) (Matrix metalloproteinase-13) (MMP-13) |
| *MSH2*** | DNA mismatch repair protein Msh2 (hMSH2) (MutS protein homolog 2) |
| *MSH6*** | DNA mismatch repair protein Msh6 (hMSH6) (G/T mismatch-binding protein) (GTBP) (GTMBP) (MutS protein homolog 6) (MutS-alpha 160 kDa subunit) (p160) |
| *MTHFR* | Methylenetetrahydrofolate reductase (EC 1.5.1.20) |
| *MTOR* | Serine/threonine-protein kinase mTOR (EC 2.7.11.1) (FK506-binding protein 12-rapamycin complex-associated protein 1) (FKBP12-rapamycin complex-associated protein) (Mammalian target of rapamycin) (mTOR) (Mechanistic target of rapamycin) (Rapamycin and FKBP12 target 1) (Rapamycin target protein 1) |
| *MUS81** | Crossover junction endonuclease MUS81 (EC 3.1.22.-) |
| *MUTYH* | Adenine DNA glycosylase (EC 3.2.2.-) (MutY homolog) (hMYH) |
| *MYC* | Myc proto-oncogene protein (Class E basic helix-loop-helix protein 39) (bHLHe39) (Proto-oncogene c-Myc) (Transcription factor p64) |
| *NF1* | Neurofibromin (Neurofibromatosis-related protein NF-1) [Cleaved into: Neurofibromin truncated] |
| *NOTCH1* | Neurogenic locus notch homolog protein 1 (Notch 1) (hN1) (Translocation-associated notch protein TAN-1) [Cleaved into: Notch 1 extracellular truncation (NEXT); Notch 1 intracellular domain (NICD)] |
| *NRAS* | GTPase NRas (Transforming protein N-Ras) |
| *PALB2** | Partner and localizer of BRCA2 |
| *PAX8* | Paired box protein Pax-8 |
| *PIK3CA* | Phosphatidylinositol 4,5-bisphosphate 3-kinase catalytic subunit alpha isoform (PI3-kinase subunit alpha) (PI3K-alpha) (PI3Kalpha) (PtdIns-3-kinase subunit alpha) (EC 2.7.1.153) (Phosphatidylinositol 4,5-bisphosphate 3-kinase 110 kDa catalytic subunit alpha) (PtdIns-3-kinase subunit p110-alpha) (p110alpha) (Phosphoinositide-3-kinase catalytic alpha polypeptide) (Serine/threonine protein kinase PIK3CA) (EC 2.7.11.1) |
| *PLCB3* | 1-phosphatidylinositol 4,5-bisphosphate phosphodiesterase beta-3 (EC 3.1.4.11) (Phosphoinositide phospholipase C-beta-3) (Phospholipase C-beta-3) (PLC-beta-3) |
| *PMS2*** | Mismatch repair endonuclease PMS2 (EC 3.1.-.-) (DNA mismatch repair protein PMS2) (PMS1 protein homolog 2) |
| *POLD1** | DNA polymerase delta catalytic subunit (EC 2.7.7.7) (EC 3.1.11.-) (DNA polymerase subunit delta p125) |
| *POLE* | DNA polymerase epsilon catalytic subunit A (EC 2.7.7.7) (DNA polymerase II subunit A) |
| *POT1* | Protection of telomeres protein 1 (hPot1) (POT1-like telomere end-binding protein) |
| *PPARG* | Peroxisome proliferator-activated receptor gamma (PPAR-gamma) (Nuclear receptor subfamily 1 group C member 3) |
| *PTEN* | Phosphatidylinositol 3,4,5-trisphosphate 3-phosphatase and dual-specificity protein phosphatase PTEN (EC 3.1.3.16) (EC 3.1.3.48) (EC 3.1.3.67) (Mutated in multiple advanced cancers 1) (Phosphatase and tensin homolog) |
| *RAD50** | DNA repair protein RAD50 (hRAD50) (EC 3.6.-.-) |
| *RAD51B** | DNA repair protein RAD51 homolog 2 (R51H2) (RAD51 homolog B) (Rad51B) (RAD51-like protein 1) |
| *RAD51C** | DNA repair protein RAD51 homolog 3 (R51H3) (RAD51 homolog C) (RAD51-like protein 2) |
| *RAD51D** | DNA repair protein RAD51 homolog 4 (R51H3) (RAD51 homolog D) (RAD51-like protein 3) (TRAD) |
| *RB1* | Retinoblastoma-associated protein (p105-Rb) (pRb) (Rb) (pp110) |
| *RET* | Proto-oncogene tyrosine-protein kinase receptor Ret (EC 2.7.10.1) (Cadherin family member 12) (Proto-oncogene c-Ret) [Cleaved into: Soluble RET kinase fragment; Extracellular cell-membrane anchored RET cadherin 120 kDa fragment] |
| *RYR3* | Ryanodine receptor 3 (RYR-3) (RyR3) (Brain ryanodine receptor-calcium release channel) (Brain-type ryanodine receptor) (Type 3 ryanodine receptor) |
| *SCG5* | Neuroendocrine protein 7B2 (Pituitary polypeptide) (Secretogranin V) (Secretogranin-5) (Secretory granule endocrine protein I) [Cleaved into: N-terminal peptide; C-terminal peptide] |
| *SMAD4* | Mothers against decapentaplegic homolog 4 (MAD homolog 4) (Mothers against DPP homolog 4) (Deletion target in pancreatic carcinoma 4) (SMAD family member 4) (SMAD 4) (Smad4) (hSMAD4) |
| *STAT3* | Signal transducer and activator of transcription 3 (Acute-phase response factor) |
| *STK11* | Serine/threonine-protein kinase STK11 (EC 2.7.11.1) (Liver kinase B1) (LKB1) (hLKB1) (Renal carcinoma antigen NY-REN-19) |
| *TGFA* | Protransforming growth factor alpha [Cleaved into: Transforming growth factor alpha (TGF-alpha) (EGF-like TGF) (ETGF) (TGF type 1)] |
| *TP53* | Cellular tumor antigen p53 (Antigen NY-CO-13) (Phosphoprotein p53) (Tumor suppressor p53) |
| *TYMS* | Thymidylate synthase (TS) (TSase) |
| *VEGFA* | Vascular endothelial growth factor A (VEGF-A) (Vascular permeability factor) (VPF) |
| *VHL* | Von Hippel-Lindau disease tumor suppressor (Protein G7) (pVHL) |
| *WNT1* | Proto-oncogene Wnt-1 (Proto-oncogene Int-1 homolog) |
| *XRCC2** | DNA repair protein XRCC2 (X-ray repair cross-complementing protein 2) |

* Genes involved in homologous recombination and ** mismatch repair pathways.

1. The UniProt Consortium. UniProt: the universal protein knowledgebase. Nucleic Acids Res 2017; 45: D158-D169.
