# Supplementary material for: Increased Levels of Genomic Instability and Mutations in Homologous Recombination Genes in Locally Advanced Rectal Carcinomas
Source: Front Oncol. 2019 May 14;9:395. doi: 10.3389/fonc.2019.00395 (PMC6527873; doi:10.3389/fonc.2019.00395)
Supplement: Supplementary file 2 [file Table_2.docx]

Supplementary Material

# Supplementary Tables

**Supplementary Table 2.** Differentially altered regions between rectal cancer cases with pathological complete (pCR N=11) or incomplete (pIR N=22) response to neoadjuvancy chemoradiotherapy.

| **Chr** | **Start** | **End** | **Type** | **p-value** | ***Ep-value** | **%pIR gain** | **%pIR loss** | **%pIR LOH** | **%pCR gain** | **%pCR loss** | **%pCR LOH** |
| --- | --- | --- | --- | --- | --- | --- | --- | --- | --- | --- | --- |
| **4** | 68345 | 600444 | loss | 0.030 | 0.638 | 4.6 | 0 | 13.6 | 0 | 27.3 | 0 |
| **4** | 600445 | 883599 | loss | 0.030 | 0.699 | 9.1 | 0 | 13.6 | 0 | 27.3 | 0 |
| **4** | 883600 | 929408 | loss | 0.030 | 0.780 | 4.6 | 0 | 13.6 | 0 | 27.3 | 0 |
| **4** | 929409 | 1055304 | loss | 0.030 | 0.750 | 9.1 | 0 | 13.6 | 0 | 27.3 | 0 |
| **4** | 1055305 | 1214772 | loss | 0.030 | 0.720 | 4.6 | 0 | 13.6 | 0 | 27.3 | 0 |
| **4** | 1214773 | 1666742 | loss | 0.030 | 0.649 | 9.1 | 0 | 13.6 | 0 | 27.3 | 0 |
| **4** | 1666743 | 1861555 | loss | 0.030 | 0.710 | 4.6 | 0 | 13.6 | 0 | 27.3 | 0 |
| **8** | 36100360 | 36280156 | loss | 0.049 | 0.712 | 9.1 | 18.2 | 4.6 | 9.1 | 54.5 | 0 |
| **8** | 37048359 | 37110223 | loss | 0.027 | 0.771 | 18.2 | 9.1 | 4.6 | 18.2 | 45.5 | 0 |
| **8** | 36280157 | 36340755 | loss | 0.049 | 0.771 | 13.6 | 18.2 | 4.6 | 9.1 | 54.5 | 0 |
| **8** | 36340756 | 36342101 | loss | 0.049 | 0.810 | 18.2 | 18.2 | 4.6 | 9.1 | 54.5 | 0 |
| **19** | 58956817 | 58956888 | gain | 0.033 | 0.886 | 4.6 | 4.6 | 4.6 | 36.4 | 0 | 9.1 |
| **20** | 26305567 | 29420337 | LOH | 0.030 | 0.298 | 45.5 | 0 | 0 | 36.4 | 9.1 | 27.3 |
| **20** | 30394518 | 31667742 | LOH | 0.030 | 0.311 | 68.2 | 0 | 0 | 72.7 | 0 | 27.3 |
| **20** | 30147144 | 30394517 | LOH | 0.030 | 0.312 | 72.7 | 0 | 0 | 72.7 | 0 | 27.3 |
| **20** | 25291777 | 25547650 | LOH | 0.030 | 0.312 | 45.5 | 9.1 | 0 | 36.4 | 27.3 | 27.3 |
| **20** | 30021951 | 30147143 | LOH | 0.030 | 0.312 | 68.2 | 0 | 0 | 72.7 | 0 | 27.3 |
| **20** | 29645544 | 30021950 | LOH | 0.030 | 0.312 | 63.6 | 0 | 0 | 72.7 | 0 | 27.3 |
| **20** | 25828321 | 26069743 | LOH | 0.030 | 0.312 | 45.5 | 0 | 0 | 36.4 | 18.2 | 27.3 |
| **20** | 29569881 | 29641279 | LOH | 0.030 | 0.313 | 59.1 | 0 | 0 | 72.7 | 0 | 27.3 |
| **20** | 26113570 | 26209739 | LOH | 0.030 | 0.313 | 45.5 | 0 | 0 | 36.4 | 18.2 | 27.3 |
| **20** | 26209740 | 26305479 | LOH | 0.030 | 0.313 | 45.5 | 0 | 0 | 45.5 | 18.2 | 27.3 |
| **20** | 25758061 | 25828320 | LOH | 0.030 | 0.313 | 45.5 | 0 | 0 | 27.3 | 18.2 | 27.3 |
| **20** | 25665141 | 25740873 | LOH | 0.030 | 0.313 | 40.9 | 0 | 0 | 36.4 | 18.2 | 27.3 |
| **20** | 29436691 | 29505815 | LOH | 0.030 | 0.313 | 50 | 0 | 0 | 54.5 | 0 | 27.3 |
| **20** | 26069744 | 26113569 | LOH | 0.030 | 0.316 | 50 | 0 | 0 | 36.4 | 18.2 | 27.3 |
| **20** | 29505816 | 29533313 | LOH | 0.030 | 0.318 | 50 | 0 | 0 | 63.6 | 0 | 27.3 |
| **20** | 26305480 | 26305566 | LOH | 0.030 | 0.318 | 45.5 | 0 | 0 | 36.4 | 18.2 | 27.3 |
| **20** | 25740874 | 25758060 | LOH | 0.030 | 0.318 | 45.5 | 0 | 0 | 36.4 | 18.2 | 27.3 |
| **20** | 25663185 | 25665140 | LOH | 0.030 | 0.318 | 36.4 | 0 | 0 | 36.4 | 18.2 | 27.3 |
| **20** | 25657485 | 25663089 | LOH | 0.030 | 0.318 | 45.5 | 0 | 0 | 36.4 | 18.2 | 27.3 |
| **20** | 25635721 | 25657484 | LOH | 0.030 | 0.318 | 45.5 | 4.6 | 0 | 36.4 | 18.2 | 27.3 |
| **20** | 25606242 | 25635720 | LOH | 0.030 | 0.318 | 40.9 | 4.6 | 0 | 36.4 | 18.2 | 27.3 |
| **20** | 25584884 | 25606241 | LOH | 0.030 | 0.318 | 45.5 | 4.6 | 0 | 36.4 | 18.2 | 27.3 |
| **20** | 29420338 | 29436690 | LOH | 0.030 | 0.318 | 50 | 0 | 0 | 54.5 | 9.1 | 27.3 |
| **20** | 25274433 | 25291776 | LOH | 0.030 | 0.318 | 40.9 | 9.1 | 0 | 36.4 | 27.3 | 27.3 |
| **20** | 29533314 | 29538641 | LOH | 0.030 | 0.318 | 45.5 | 0 | 0 | 63.6 | 0 | 27.3 |
| **20** | 25265985 | 25274432 | LOH | 0.030 | 0.318 | 36.4 | 9.1 | 0 | 36.4 | 27.3 | 27.3 |
| **20** | 29538642 | 29544849 | LOH | 0.030 | 0.318 | 50 | 0 | 0 | 63.6 | 0 | 27.3 |
| **20** | 29544850 | 29563270 | LOH | 0.030 | 0.318 | 54.5 | 0 | 0 | 63.6 | 0 | 27.3 |
| **20** | 29563271 | 29569880 | LOH | 0.030 | 0.318 | 59.1 | 0 | 0 | 63.6 | 0 | 27.3 |
| **20** | 29641280 | 29645543 | LOH | 0.030 | 0.318 | 54.5 | 0 | 0 | 72.7 | 0 | 27.3 |
| **20** | 25663090 | 25663184 | LOH | 0.030 | 0.318 | 40.9 | 0 | 0 | 36.4 | 18.2 | 27.3 |
| **20** | 25547651 | 25584883 | LOH | 0.030 | 0.318 | 45.5 | 4.6 | 0 | 36.4 | 27.3 | 27.3 |
| **21** | 35020505 | 40248388 | loss | 0.031 | 0.319 | 9.1 | 36.4 | 9.1 | 9.1 | 0 | 9.1 |

***** empirical p-values for permutation testing
