# Supplementary material for: Increased Levels of Genomic Instability and Mutations in Homologous Recombination Genes in Locally Advanced Rectal Carcinomas
Source: Front Oncol. 2019 May 14;9:395. doi: 10.3389/fonc.2019.00395 (PMC6527873; doi:10.3389/fonc.2019.00395)
Supplement: Supplementary file 3 [file Table_3.DOCX]

Supplementary Material

# Supplementary Tables

**Supplementary Table S3.** Genomic Instability Index (GII) and homologous recombination deficiency (HRD) scores (tAI, LOH and LST) of rectal cancer patients according to response.

| **Cases** | **GII^a^** | **LST^b^** | **HRD-LOH^c^** | **tAI^d^** |
| --- | --- | --- | --- | --- |
| ***Pathological Complete Response - pCR*** | | | | |
| **RET10** | **0.475** | 3 | 1 | 4 |
| **RET11** | **0.531** | 4 | 3 | 9 |
| **RET13** | 0.062 | **26** | 0 | **18** |
| **RET28** | **0.494** | 3 | 5 | 13 |
| **RET29** | **0.852** | 12 | 9 | **18** |
| **RET33** | **0.572** | **20** | 3 | **15** |
| **RET34** | 0.245 | 10 | **11** | 9 |
| **RET41** | 0.179 | 2 | 1 | 5 |
| **RET42** | 0.000 | 0 | 0 | 8 |
| **RET43** | **0.949** | 12 | **11** | **15** |
| **RET44** | **0.397** | 12 | **15** | 13 |
| ***Pathological Incomplete Response - pIR*** | | | | |
| **RET2** | **0.392** | 13 | 0 | **15** |
| **RET3** | 0.137 | 1 | 0 | 7 |
| **RET4** | **0.488** | 3 | 0 | **16** |
| **RET5** | 0.218 | 5 | 7 | 8 |
| **RET6** | 0.283 | 1 | 2 | 9 |
| **RET7** | 0.256 | 3 | 5 | 3 |
| **RET8** | 0.184 | 13 | **11** | **14** |
| **RET9** | **0.431** | 4 | 9 | **14** |
| **RET12** | **0.647** | 14 | 2 | **23** |
| **RET15** | **0.663** | 14 | 0 | **26** |
| **RET17** | **0.480** | 12 | 1 | **23** |
| **RET18** | **0.585** | 5 | **12** | **17** |
| **RET19** | 0.038 | 13 | 0 | **21** |
| **RET20** | 0.283 | 4 | 3 | 12 |
| **RET21** | **0.305** | 7 | 1 | **15** |
| **RET24** | **0.479** | 8 | 0 | 8 |
| **RET25** | **0.479** | 6 | 5 | **17** |
| **RET26** | 0.033 | 10 | 0 | **26** |
| **RET27** | 0.222 | 5 | 7 | **15** |
| **RET30** | 0.065 | 1 | 1 | 3 |
| **RET31** | **0.358** | 10 | 0 | **23** |
| **RET32** | 0.000 | 1 | 0 | 4 |

Thresholds: **a:** >0.286; **b:** > 15 for 2N, >20 for 4N; **c:** > 10; **d:** ≥ 14. Bold cases present high values.
