# Supplementary material for: Increased Levels of Genomic Instability and Mutations in Homologous Recombination Genes in Locally Advanced Rectal Carcinomas
Source: Front Oncol. 2019 May 14;9:395. doi: 10.3389/fonc.2019.00395 (PMC6527873; doi:10.3389/fonc.2019.00395)
Supplement: Supplementary file 4 [file Table_4.DOCX]

Supplementary Material

# Supplementary Tables

**Supplementary Table S4**. Mutational profile found in 31 locally advanced rectal cancer cases.

| **Case** | **Gene** | **Annotation** | **HGVS c** | **HGVS p** | **Response** |
| --- | --- | --- | --- | --- | --- |
| **RET2** | *APC* | splice_donor_variant& intron_variant | c.1548+2T>C | . | pIR |
|  | *AR* | missense_variant | c.2521C>A | p.Arg841Ser |  |
|  | *ERBB3* | missense_variant | c.695C>T | p.Ala232Val |  |
|  | *POLD1* | missense_variant | c.2275G>A | p.Val759Ile |  |
|  | *TP53* | missense_variant | c.818G>A | p.Arg273His |  |
| **RET3** | *APC* | stop_gained | c.4189G>T | p.Glu1397* | pIR |
|  | *APC* | stop_gained | c.2413C>T | p.Arg805* |  |
|  | *IGF1R* | intragenic_variant | n.99501823G>A | . |  |
|  | *KRAS* | missense_variant | c.38G>A | p.Gly13Asp |  |
|  | *MLH3* | 3_prime_UTR_variant | c.*1237A>G | . |  |
|  | *TP53* | protein_protein_contact | c.817C>T | p.Arg273Cys |  |
| **RET4** | *APC* | frameshift_variant | c.4435delG | p.Val1479fs | pIR |
|  | *APC* | stop_gained | c.4057G>T | p.Glu1353* |  |
|  | *EZH2* | intron_variant | c.246+15C>G | . |  |
|  | *TP53* | splice_acceptor_variant&intron_variant | c.783-1G>A | . |  |
| **RET5** | *APC* | stop_gained | c.3880C>T | p.Gln1294* | pIR |
|  | *BRIP1* | missense_variant | c.517C>T | p.Arg173Cys |  |
|  | *FBXW7* | stop_gained | c.286G>T | p.Glu96* |  |
|  | *FLT1* | intron_variant | c.1970-1266C>A | . |  |
|  | *KRAS* | missense_variant | c.34G>A | p.Gly12Ser |  |
|  | *TP53* | protein_protein_contact | c.811G>A | p.Glu271Lys |  |
| **RET6** | *ERBB2* | missense_variant | c.3557C>A | p.Ala1186Asp | pIR |
|  | *HGF* | protein_protein_contact | c.706G>T | . |  |
|  | *KRAS* | missense_variant | c.35G>A | p.Gly12Asp |  |
|  | *MMP1* | missense_variant | c.755A>G | p.Asp252Gly |  |
|  | *RYR3* | intragenic_variant | n.33993250C>T | . |  |
|  | *TP53* | inframe_insertion | c.1009_1010ins ACTTCGAGATGTTCGTGGGCGTGA | p.Arg337delinsHisPheGluMetPheValGlyValSer |  |
|  | *TP53* | frameshift_variant | c.1011_1012insAC | p.Phe338fs |  |
| **RET7** | *APC* | stop_gained | c.3916G>T | p.Glu1306* | pIR |
|  | *APC* | frameshift_variant& stop_gained | c.1499dupA | p.Tyr500fs |  |
|  | *ATM* | stop_gained | c.3663G>A | p.Trp1221* |  |
|  | *ATM* | missense_variant | c.5438T>C | p.Phe1813Ser |  |
|  | *FLT1* | intron_variant | c.3636-15_3636-14delCT | . |  |
|  | *KRAS* | protein_protein_contact | c.436G>A | . |  |
|  | *PIK3CA* | missense_variant | c.3103G>A | p.Ala1035Thr |  |
| **RET8** | *BMPR1A* | stop_gained | c.1436T>A | p.Leu479* | pIR |
|  | *COL11A1* | missense_variant | c.130G>A | p.Ala44Thr |  |
|  | *ERBB4* | 5_prime_UTR_variant | c.-92C>T | . |  |
|  | *KRAS* | missense_variant | c.35G>A | p.Gly12Asp |  |
|  | *MMP1* | stop_gained | c.1389G>A | p.Trp463* |  |
|  | *PIK3CA* | missense_variant | c.1637A>C | p.Gln546Pro |  |
|  | *SCG5* | missense_variant | c.130C>T | p.Leu44Phe |  |
|  | *TP53* | missense_variant | c.524G>A | p.Arg175His |  |
| **RET9** | *APC* | stop_gained | c.4012C>T | p.Gln1338* | pIR |
|  | *FGFR2* | 5_prime_UTR_variant | c.-61G>T | . |  |
|  | *TP53* | protein_protein_contact | c.641_646dupATAGTG | p.Val218fs |  |
| **RET10** | *APC* | frameshift_variant | c.1847dupT | p.Leu616fs | pCR |
|  | *APC* | splice_region_variant& synonymous_variant | c.1548G>A | p.Lys516Lys |  |
|  | *ERBB2* | missense_variant | c.2434G>A | p.Val812Ile |  |
|  | *KRAS* | missense_variant | c.35G>A | p.Gly12Asp |  |
| **RET11** | *APC* | frameshift_variant | c.3323dupA | p.Asn1108fs | pCR |
|  | *APC* | stop_gained | c.4067C>G | p.Ser1356* |  |
|  | *APC* | intragenic_variant | n.112043263C>T | . |  |
|  | *ERBB3* | missense_variant | c.2737G>A | p.Ala913Thr |  |
|  | *TP53* | protein_protein_contact | c.463A>C | p.Thr155Pro |  |
| **RET12** | *APC* | frameshift_variant | c.4468delC | p.His1490fs | pIR |
|  | *CCND1* | 3_prime_UTR_variant | c.*2066A>G | . |  |
|  | *COL11A1* | missense_variant | c.3208T>A | p.Ser1070Thr |  |
|  | *DICER1* | missense_variant | c.2201C>G | p.Thr734Ser |  |
|  | *RAD51C* | missense_variant | c.859A>G | p.Thr287Ala |  |
|  | *TP53* | frameshift_variant | c.614_615delAT | p.Tyr205fs |  |
| **RET13** | *APC* | frameshift_variant | c.4209delC | p.Ser1404fs | pCR |
|  | *APC* | stop_gained & disruptive_inframe_deletion | c.4211_4216delCCGTTC | p.Ser1404_Gln1406delinsTer |  |
|  | *MYC* | missense_variant | c.1150G>A | p.Glu384Lys |  |
| **RET15** | *KRAS* | protein_protein_contact | c.436G>A | . | pIR |
|  | *PIK3CA* | missense_variant | c.1624G>A | p.Glu542Lys |  |
|  | *SMAD4* | missense_variant | c.1081C>A | p.Arg361Ser |  |
|  | *TP53* | missense_variant | c.742C>T | p.Arg248Trp |  |
| **RET17** | *APC* | stop_gained | c.3340C>T | p.Arg1114* | pIR |
|  | *APC* | stop_gained | c.4037C>A | p.Ser1346* |  |
|  | *FLT1* | intron_variant | c.1969+1608G>A | . |  |
|  | *KRAS* | missense_variant | c.35G>A | p.Gly12Asp |  |
|  | *MTOR* | missense_variant | c.5930C>A | p.Thr1977Lys |  |
|  | *MTOR* | missense_variant | c.3461G>A | p.Arg1154Gln |  |
|  | *PIK3CA* | missense_variant | c.1637A>C | p.Gln546Pro |  |
|  | *RAD51C* | missense_variant | c.859A>G | p.Thr287Ala |  |
| **RET18** | *APC* | frameshift_variant | c.4245delT | p.Ser1415fs | pIR |
|  | *ARF1* | 3_prime_UTR_variant | c.*1020G>A | . |  |
|  | *ARF1* | 3_prime_UTR_variant | c.*1105G>A | . |  |
|  | *KRAS* | missense_variant | c.35G>T | p.Gly12Val |  |
|  | *TP53* | missense_variant | c.841G>T | p.Asp281Tyr |  |
|  | *TP53* | protein_protein_contact | c.832C>T | p.Pro278Ser |  |
|  | *WNT1* | 3_prime_UTR_variant | c.*654delC | . |  |
| **RET19** | *TP53* | frameshift_variant | c.201_202insTT | p.Glu68fs | pIR |
| **RET20** | *APC* | frameshift_variant | c.4668delT | p.Ile1557fs | pIR |
|  | *APC* | stop_gained | c.266C>A | p.Ser89* |  |
|  | *APC* | stop_gained | c.2269C>T | p.Gln757* |  |
|  | *ERBB3* | missense_variant | c.2000G>A | p.Arg667His |  |
|  | *FLT4* | splice_acceptor_variant& splice_region_variant& intron_variant | c.3220-35_3220-2del TCCACCACGGGACAAGCTTCCCTCTGTCTCCCCA | . |  |
|  | *MSH6* | missense_variant | c.274C>A | p.Pro92Thr |  |
|  | *NoCH1* | missense_variant | c.4028C>T | p.Ala1343Val |  |
|  | *PIK3CA* | missense_variant | c.1624G>A | p.Glu542Lys |  |
|  | *TP53* | missense_variant | c.642T>G | p.His214Gln |  |
|  | *TP53* | splice_donor_variant& intron_variant | c.782+1G>T | . |  |
|  | *WNT1* | 3_prime_UTR_variant | c.*654delC | . |  |
| **RET24** | *APC* | stop_gained | c.1787C>A | p.Ser596* | pIR |
|  | *APC* | frameshift_variant | c.4245delT | p.Ser1415fs |  |
|  | *FLT4* | splice_acceptor_variant& splice_region_variant& intron_variant | c.3220-35_3220-2del TCCACCACGGGACAAGCTTCCCTCTGTCTCCCCA | . |  |
|  | *TP53* | missense_variant | c.645T>A | p.Ser215Arg |  |
|  | *WNT1* | missense_variant | c.1007C>T | p.Thr336Met |  |
| **RET25** | *APC* | stop_gained& splice_region_variant | c.646C>T | p.Arg216* | pIR |
|  | *POLE* | missense_variant | c.1007A>G | p.Asn336Ser |  |
|  | *RYR3* | intragenic_variant | n.34157570_34157585dupATTTTGAAATTGATTT | . |  |
|  | *TP53* | stop_gained | c.493C>T | p.Gln165* |  |
| **RET26** | *APC* | stop_gained | c.2626C>T | p.Arg876* | pIR |
|  | *JUN* | 5_prime_UTR_variant | c.-826C>T | . |  |
|  | *NF1* | missense_variant | c.6773G>A | p.Arg2258Gln |  |
|  | *TP53* | splice_acceptor_variant& intron_variant | c.673-2A>T | . |  |
| **RET27** | *APC* | stop_gained | c.3544A>T | p.Lys1182* | pIR |
|  | *BRAF* | missense_variant | c.1781A>G | p.Asp594Gly |  |
|  | *FBXW7* | frameshift_variant | c.235delG | p.Glu79fs |  |
|  | *FBXW7* | frameshift_variant& splice_region_variant | c.494_500dup CAACAAA | p.Lys167fs |  |
|  | *FLT1* | missense_variant | c.1700C>T | p.Pro567Leu |  |
|  | *FLT4* | missense_variant | c.2563G>A | p.Ala855Thr |  |
|  | *IDH1* | protein_protein_contact | c.301A>T | . |  |
|  | *MTHFR* | stop_gained | c.233C>G | p.Ser78* |  |
|  | *SMAD4* | protein_protein_contact | c.1054G>A | . |  |
|  | *TP53* | missense_variant | c.722C>T | p.Ser241Phe |  |
| **RET28** | *ABCC1* | 3_prime_UTR_variant | c.*1015_*1016delGC | . | pCR |
|  | *APC* | frameshift_variant | c.3900_3909del TACCCTGCAA | p.Asn1300fs |  |
|  | *APC* | missense_variant | c.2513G>C | p.Arg838Thr |  |
|  | *APC* | missense_variant | c.6736G>A | p.Val2246Ile |  |
|  | *DICER1* | intron_variant | c.-45-17C>A | . |  |
|  | *ERBB4* | stop_gained | c.2512C>T | p.Arg838* |  |
|  | *RAD51B* | missense_variant | c.728A>G | p.Lys243Arg |  |
|  | *TP53* | protein_protein_contact | c.814G>T | p.Val272Leu |  |
| **RET29** | *DICER1* | missense_variant | c.5536T>G | p.Ser1846Ala | pCR |
|  | *NF1* | frameshift_variant | c.3051delA | p.Gln1017fs |  |
|  | *PIK3CA-KCNMB3* | intergenic_region | n.178956145A>C | . |  |
|  | *PIK3CA-KCNMB3* | intergenic_region | n.178957276_178957280delTTAAC | . |  |
|  | *RYR3* | intragenic_variant | n.33795814C>G | . |  |
|  | *TP53* | splice_acceptor_variant& intron_variant | c.783-1G>T | . |  |
| **RET30** | *APC* | stop_gained | c.2269C>T | p.Gln757* | pIR |
|  | *APC* | frameshift_variant | c.4473dupT | p.Ala1492fs |  |
|  | *ATM* | missense_variant | c.6067G>A | p.Gly2023Arg |  |
|  | *FBXW7* | stop_gained | c.1972C>T | p.Arg658* |  |
|  | *JUN* | inframe_deletion | c.805_819delTGCCGAAAAAGGAAG | p.Cys269_Lys273del |  |
|  | *KRAS* | missense_variant | c.351A>C | p.Lys117Asn |  |
|  | *MUTYH* | missense_variant& splice_region_variant | c.1187G>A | p.Gly396Asp |  |
|  | *RYR3* | intragenic_variant | n.34158080G>C | . |  |
|  | *TYMS* | protein_protein_contact | c.13G>A | . |  |
| **RET31** | *APC* | intragenic_variant | n.112043263C>T | . | pIR |
|  | *APC* | stop_gained | c.4012C>T | p.Gln1338* |  |
|  | *MTHFR* | missense_variant | c.1409A>T | p.Glu470Val |  |
|  | *PTEN* | intragenic_variant | n.89692944G>T | . |  |
|  | *TP53* | protein_protein_contact | c.704_709delACTACA | . |  |
| **RET32** | *APC* | frameshift_variant | c.4473dupT | p.Ala1492fs | pIR |
|  | *APC* | stop_gained | c.2821G>T | p.Glu941* |  |
|  | *COL11A1* | missense_variant | c.4454G>A | p.Arg1485Gln |  |
|  | *EGF* | missense_variant | c.2094G>C | p.Trp698Cys |  |
|  | *FBXW7* | missense_variant | c.1394G>A | p.Arg465His |  |
|  | *FLT4* | missense_variant | c.1310G>A | p.Arg437His |  |
|  | *FLT4* | missense_variant | c.151T>C | p.Cys51Arg |  |
|  | *KRAS* | missense_variant | c.35G>A | p.Gly12Asp |  |
|  | *MSH6* | splice_acceptor_variant& splice_region_variant& intron_variant | c.3802-7_3802-4delTCTT | . |  |
|  | *MSH6* | protein_protein_contact | c.3226C>T | . |  |
|  | *TP53* | stop_gained | c.637C>T | p.Arg213* |  |
|  | *TP53* | missense_variant | c.524G>A | p.Arg175His |  |
| **RET33** | *APC* | stop_gained | c.2413C>T | p.Arg805* | pCR |
|  | *APC* | stop_gained | c.3856G>T | p.Glu1286* |  |
|  | *KRAS* | missense_variant | c.35G>T | p.Gly12Val |  |
|  | *RYR3* | intragenic_variant | n.33988566C>T | . |  |
|  | *TP53* | missense_variant | c.659A>G | p.Tyr220Cys |  |
| **RET34** | *APC* | stop_gained | c.2674G>T | p.Glu892* | pCR |
|  | *AR* | missense_variant | c.88G>A | p.Val30Met |  |
|  | *DNMT3A* | missense_variant | c.89A>C | p.Glu30Ala |  |
|  | *KRAS* | missense_variant | c.38G>A | p.Gly13Asp |  |
|  | *MLH1* | missense_variant | c.394G>C | p.Asp132His |  |
|  | *MTOR* | missense_variant | c.2563G>A | p.Val855Ile |  |
|  | *RYR3* | intragenic_variant | n.33941351C>T | . |  |
|  | *TP53* | protein_protein_contact | c.427G>A | p.Val143Met |  |
| **RET41** | *ABCG2* | missense_variant | c.221G>A | p.Gly74Asp | pCR |
|  | *APC* | stop_gained | c.4099C>T | p.Gln1367* |  |
|  | *TP53* | missense_variant | c.578A>T | p.His193Leu |  |
| **RET43** | *ALK* | missense_variant | c.592G>A | p.Val198Met | pCR |
|  | *APC* | frameshift_variant | c.4364delA | p.Asn1455fs |  |
|  | *APC* | missense_variant | c.4318C>T | p.Pro1440Ser |  |
|  | *KRAS* | missense_variant | c.35G>C | p.Gly12Ala |  |
|  | *MAP2K1* | 3_prime_UTR_variant | c.*843T>G | . |  |
|  | *TP53* | missense_variant | c.524G>A | p.Arg175His |  |
| **RET44** | *MMP1* | splice_donor_variant& intron_variant | c.105+2T>C | . | pCR |
|  | *TP53* | missense_variant | c.742C>T | p.Arg248Trp |  |

HGVS: Human Genome Variation Society; c. represents the coding sequence position and p., the amino acid position according to RefSeq (<http://varnomen.hgvs.org/>); pIR: incomplete response; pCR complete response
