# Supplementary material for: Increased Levels of Genomic Instability and Mutations in Homologous Recombination Genes in Locally Advanced Rectal Carcinomas
Source: Front Oncol. 2019 May 14;9:395. doi: 10.3389/fonc.2019.00395 (PMC6527873; doi:10.3389/fonc.2019.00395)
Supplement: Supplementary file 5 [file Data_Sheet_1.docx]

Supplementary Material

# Supplementary Figures


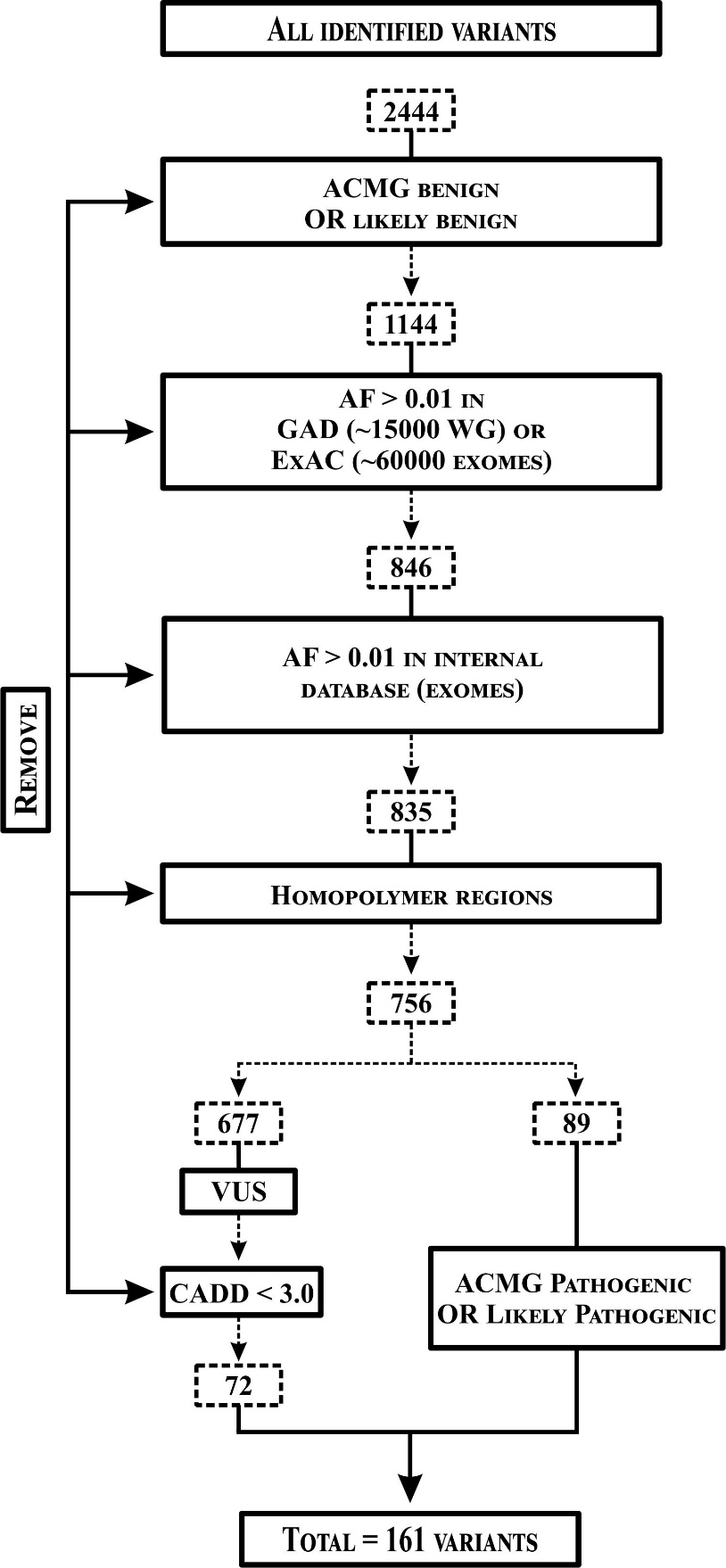


**Supplementary Figure S1.** Variant filtering pipeline. Benign or likely benign variants classified according to the American College of Medical Genetics and Genomics (**ACMG**) were excluded. Subsequently, variants with allele frequency (**AF**) > 0.01 in the GAD [1] and/or in the ExAC [2] were also excluded from further analysis. The remaining variants were manually curated using the Genome Browse software (Golden Helix Inc., Bozeman, MT). The variants mapped in homopolymer regions were excluded and a new filtering was applied to exclude variants of uncertain significance (VUS, classified by ACMG) with CADD score < 3.0.

1. Becker KG, Barnes KC, Bright TJ, Wang SA. The genetic association database. Nat Genet 2004; 36: 431-432.

2. Lek M, Karczewski KJ, Minikel EV et al. Analysis of protein-coding genetic variation in 60,706 humans. Nature 2016; 536: 285-291.
