# Supplementary material for: Increased Levels of Genomic Instability and Mutations in Homologous Recombination Genes in Locally Advanced Rectal Carcinomas
Source: Front Oncol. 2019 May 14;9:395. doi: 10.3389/fonc.2019.00395 (PMC6527873; doi:10.3389/fonc.2019.00395)
Supplement: Supplementary file 6 [file Data_Sheet_2.docx]

Supplementary Material

# Supplementary Figures


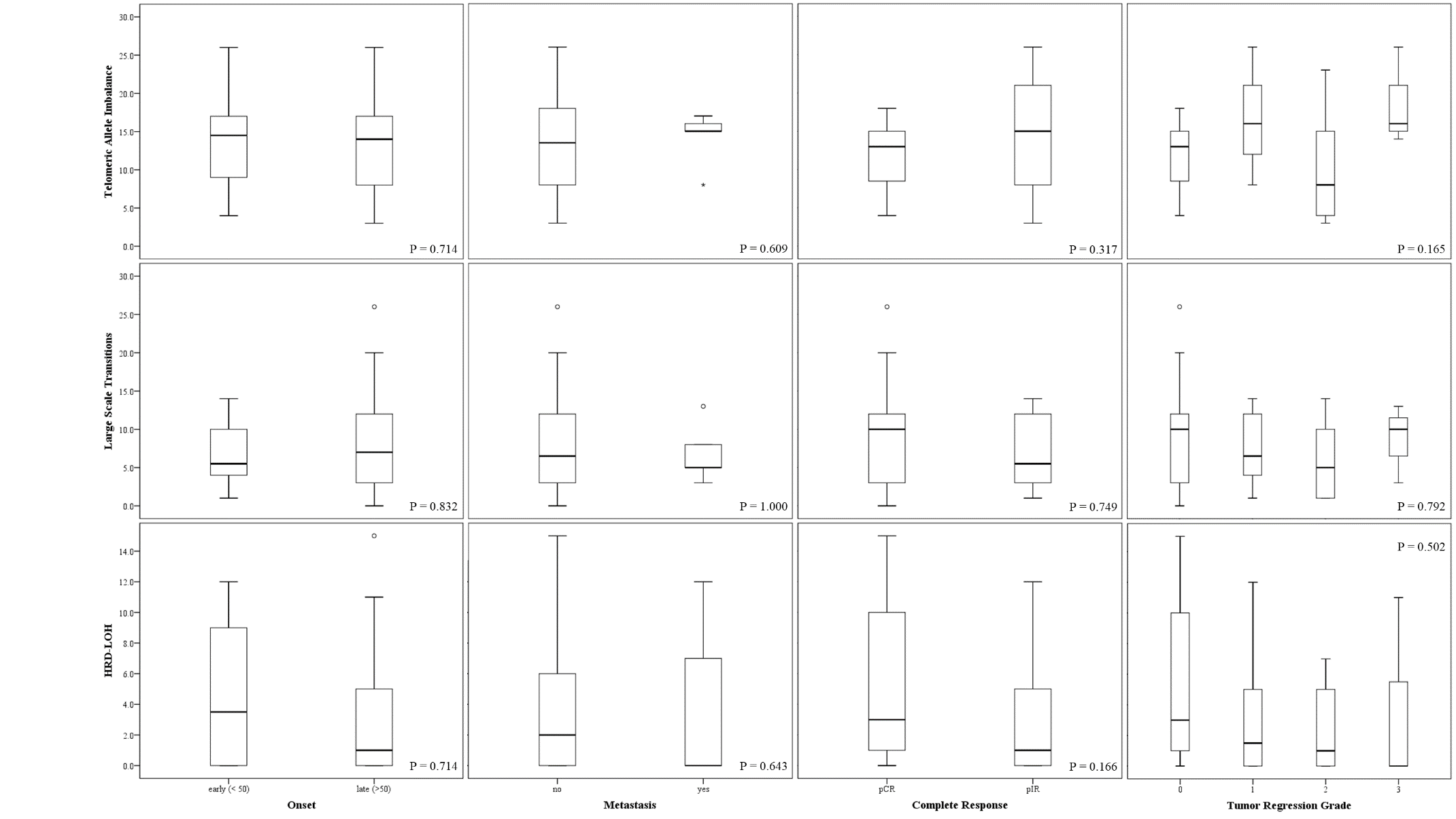
**A**

**B**

**C**

**Supplementary Figure S2**. Homologous Recombination Deficiency Scores (**A:** tAI, **B:** LST and **C:** HRD-LOH) in rectal cancer according to clinical characteristics.
