# Supplementary material for: Increased Levels of Genomic Instability and Mutations in Homologous Recombination Genes in Locally Advanced Rectal Carcinomas
Source: Front Oncol. 2019 May 14;9:395. doi: 10.3389/fonc.2019.00395 (PMC6527873; doi:10.3389/fonc.2019.00395)
Supplement: Supplementary file 7 [file Data_Sheet_3.docx]

Supplementary Material

# Supplementary Figures

**TCGA (N=145)**

**Our data (N= 31)**


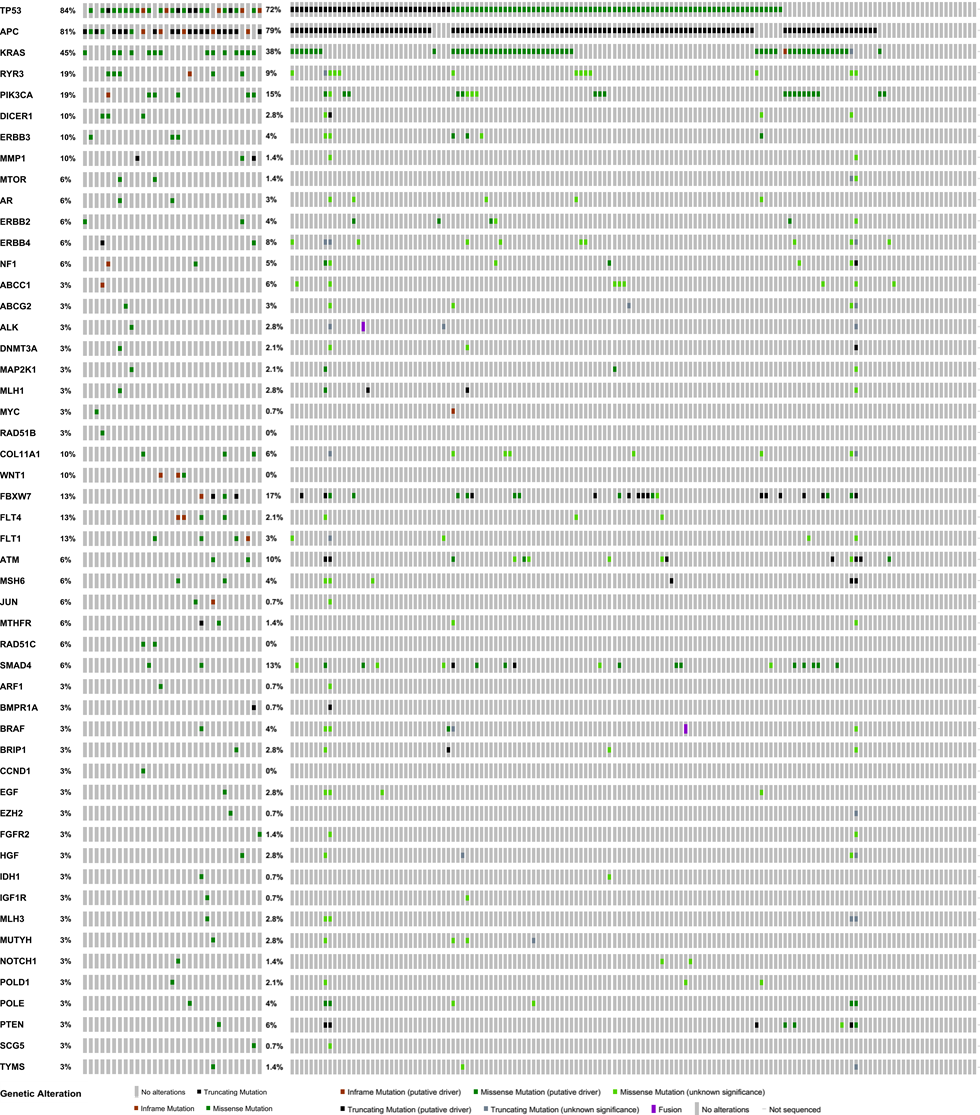


**Supplementary Figure S3.** Frequency of mutations in 51 genes in rectal cancer. Left side, our 31 cases analyzed by targeted-NGS; right side, 145 cases from TCGA Colorectal Adenocarcinoma (TCGA, Nature 2012) and Rectum Adenocarcinoma (TCGA, PanCancer Atlas).
